# Supplementary material for: The Secret World of Shrimps: Polarisation Vision at Its Best
Source: PLoS One. 2008 May 14;3(5):e2190. doi: 10.1371/journal.pone.0002190 (PMC2377063; doi:10.1371/journal.pone.0002190)
Supplement: Table S1 — Overview of recordings (0.11 MB DOC) [file pone.0002190.s001.doc]

**Table S1:** Overview of recordings

**Hemispheres** polarisation

| *Area* | *Group* | *Cell* | *Eye* | $10^{\delta}$ | $v$ | $h$ | $d$ | $a$ | $r$ | $l$ | $S_{1}$ | $S_{2}$ | $S_{3}$ | $P$ | $D$ |
| --- | --- | --- | --- | --- | --- | --- | --- | --- | --- | --- | --- | --- | --- | --- | --- |
| *DH | I | R1 | right | 9.44 | 41.3 | 42.6 | 30.0 | 44.0 | 41.6 | 41.6 | -0.015 | -0.189 | 0.000 | 0.190 | 0.99 |
| DH | II | R6 | right | 3.20 | 24.9 | 26.2 | 30.0 | 22.0 | 26.1 | 25.8 | -0.025 | 0.154 | 0.006 | 0.156 | 0.97 |
| DH | I | R4 | right | 5.26 | 35.4 | 38.1 | 29.6 | 39.0 | 36.4 | 36.2 | -0.037 | -0.137 | 0.003 | 0.142 | 0.93 |
| DH | I | R5 | right | 10.00 | 43.8 | 43.8 | 35.2 | 47.6 | 42.8 | 43.2 | 0.000 | -0.150 | -0.005 | 0.150 | 1.00 |
| DH | II | R2 | right | 7.40 | 43.9 | 42.2 | 45.6 | 36.8 | 43.2 | 41.6 | 0.017 | 0.107 | 0.019 | 0.110 | 0.95 |
| DH | II | R7 | left | 2.44 | 40.8 | 41.4 | 41.6 | 35.2 | 40.0 | 41.2 | -0.007 | 0.083 | -0.015 | 0.085 | 0.96 |
| DH | I | R5 | right | 3.90 | 39.4 | 38.8 | 33.5 | 41.6 | 38.2 | 38.2 | 0.008 | -0.108 | 0.000 | 0.108 | 0.99 |
| *DH | II | R3 | right | 5.60 | 39.8 | 40.0 | 42.4 | 31.2 | 39.2 | 38.0 | -0.002 | 0.152 | 0.016 | 0.153 | 0.99 |
| *VH | I | R1 | right | 10.56 | 26.8 | 39.9 | 35.2 | 36.0 | 36.7 | 35.8 | -0.196 | -0.012 | 0.012 | 0.197 | 0.99 |
| *VH | II | R7 | right | 5.40 | 40.8 | 29.6 | 35.0 | 36.0 | 32.4 | 32.0 | 0.159 | -0.014 | 0.006 | 0.160 | 0.99 |
| ***Mean*** |  |  |  | **6.32** |  |  |  |  |  |  |  |  |  | **0.145** | **0.98** |
| ***sd*** |  |  |  | **2.75** |  |  |  |  |  |  |  |  |  | **0.036** | **0.02** |

**Mid-band** polarisation

| *Area* | *Group* | *Cell* | *Eye* | $10^{\delta}$ | $v$ | $h$ | $d$ | $a$ | $r$ | $l$ | $S_{1}$ | $S_{2}$ | $S_{3}$ | $P$ | $D$ |
| --- | --- | --- | --- | --- | --- | --- | --- | --- | --- | --- | --- | --- | --- | --- | --- |
| row 6 | I | R5 | right | 5.14 | 36.8 | 32.6 | 33.8 | 35.2 | 41.2 | 19.2 | 0.061 | -0.020 | 0.364 | 0.370 | 0.99 |
| *row 5 | I | R1 | right | 10.84 | 45.3 | 43.1 | 44.4 | 43.1 | 48.7 | 19.2 | 0.025 | 0.015 | 0.434 | 0.435 | 0.97 |
| row 6 | II | R3 | right | 12.59 | 39.2 | 41.2 | 39.8 | 40.2 | 21.6 | 50.0 | -0.025 | -0.005 | -0.397 | 0.397 | 0.93 |
| row 6 | I | R4 | left | 11.65 | 42.0 | 41.6 | 41.9 | 41.7 | 27.4 | 45.4 | 0.005 | 0.003 | -0.247 | 0.247 | 1.00 |
| row 5 | I | R4 | right | 4.80 | 36.8 | 33.2 | 34.4 | 34.9 | 35.8 | 20.8 | 0.051 | -0.007 | 0.265 | 0.270 | 0.95 |
| row 5 | II | R2 | left | 10.00 | 38.2 | 38.2 | 38.2 | 38.2 | 40.0 | 18.0 | 0.000 | 0.000 | 0.379 | 0.379 | 0.96 |
| row 5 | I | R5 | left | 3.90 | 33.6 | 33.6 | 33.5 | 33.4 | 20.2 | 39.4 | 0.000 | 0.001 | -0.322 | 0.322 | 0.99 |
| row 6 | I | R1 | left | 12.43 | 46.4 | 44.0 | 42.3 | 44.8 | 25.4 | 50.2 | 0.027 | -0.029 | -0.328 | 0.330 | 0.99 |
| row 5 | I | R1 | left | 14.33 | 40.8 | 40.8 | 40.6 | 40.9 | 23.6 | 44.3 | 0.000 | -0.004 | -0.305 | 0.305 | 0.99 |
| ***Mean*** |  |  |  | **9.52** |  |  |  |  |  |  |  |  |  | **0.340** | **0.99** |
| ***sd*** |  |  |  | **3.88** |  |  |  |  |  |  |  |  |  | **0.061** | **0.01** |

10, polarisation sensitivity; $v,h,d,a,r,l$, peak responses of photoreceptors (mV) to the respective polarisation stimuli; $S_{i}$, derived Stokes’ parameters; $P$, degree of polarisation; $D$, discrimination of preferred polarisation. Entries indicated with * are shown in Figure 4 (main text) and Figure S2 (supporting information).
