# Supplementary figures and images for: The Secret World of Shrimps: Polarisation Vision at Its Best
Source: PLoS One. 2008 May 14;3(5):e2190. doi: 10.1371/journal.pone.0002190 (PMC2377063; doi:10.1371/journal.pone.0002190)

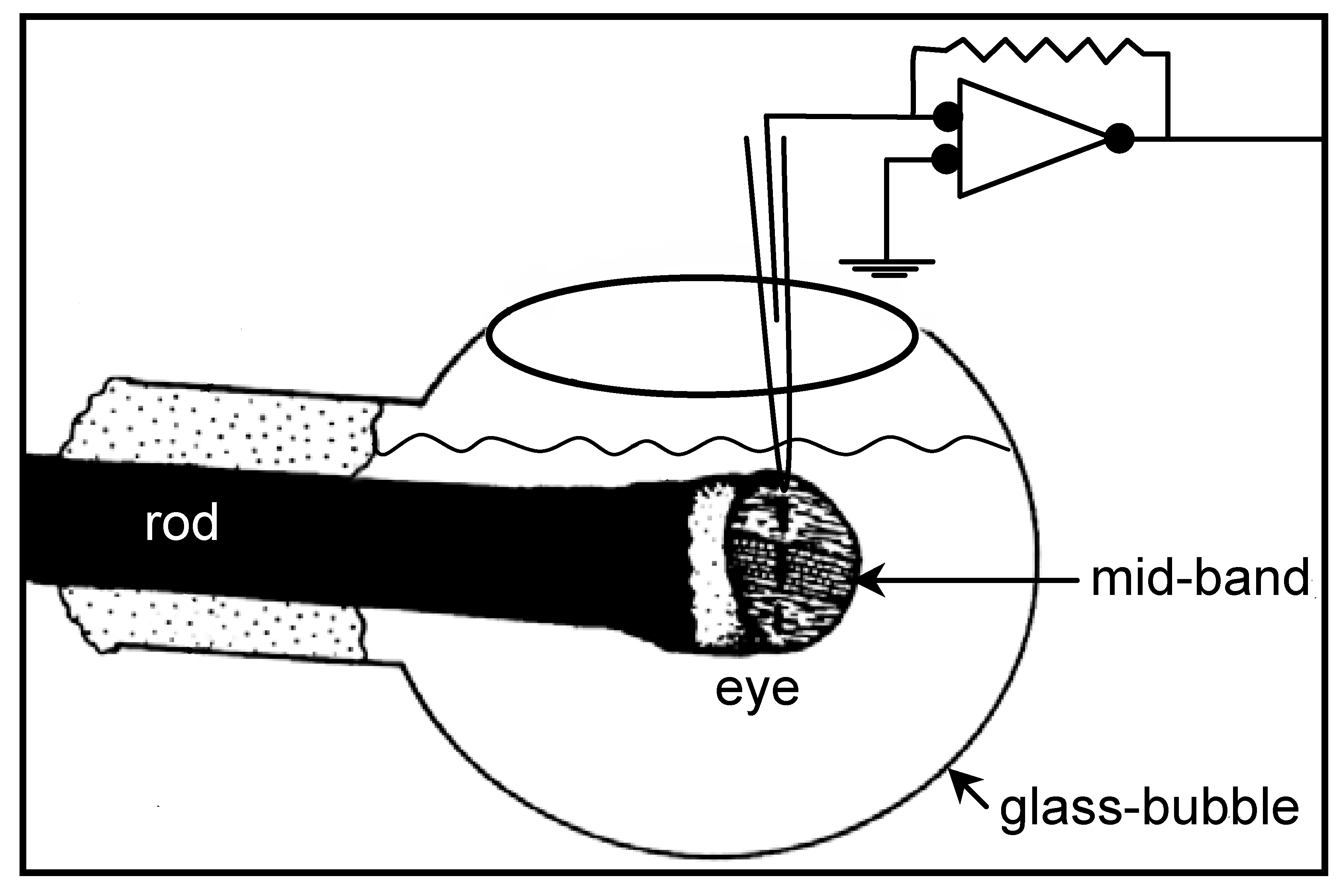

Supplement: Figure S1 — Preparation for electrophysiological recordings. The isolated eye was mounted on a plastic rod and placed into a glass-bubble filled with stomatopod saline so that the lateral mid-band was oriented horizontally. The intracellular electrode was lowered vertically through a small hole cut into the lateral cornea of the dorsal hemisphere. After Kleinlogel and Marshall (2006). (0.73 MB TIF) [file pone.0002190.s002.tif]
